# Semantics-based plausible reasoning to extend the knowledge coverage of medical knowledge bases for improved clinical decision support

**DOI:** 10.1186/s13040-017-0123-y

**Published:** 2017-02-10

**Authors:** Hossein Mohammadhassanzadeh, William Van Woensel, Samina Raza Abidi, Syed Sibte Raza Abidi

**Affiliations:** 1 NICHE Research Group, Faculty of Computer Science, Dalhousie University, Halifax, NS B3H4R2 Canada; 2 Medical Informatics, Faculty of Medicine, Dalhousie University, Halifax, NS B3H4R2 Canada

**Keywords:** Medical knowledge bases, Semantic Web reasoning, Plausible reasoning, Inductive generalization, Analogical reasoning

## Abstract

**Background:**

Capturing complete medical knowledge is challenging-often due to incomplete patient Electronic Health Records (EHR), but also because of valuable, tacit medical knowledge hidden away in physicians’ experiences. To extend the coverage of incomplete medical knowledge-based systems beyond their deductive closure, and thus enhance their decision-support capabilities, we argue that innovative, multi-strategy reasoning approaches should be applied. In particular, *plausible reasoning* mechanisms apply patterns from human thought processes, such as generalization, similarity and interpolation, based on attributional, hierarchical, and relational knowledge. Plausible reasoning mechanisms include *inductive reasoning*, which generalizes the commonalities among the data to induce new rules, and *analogical reasoning*, which is guided by data similarities to infer new facts. By further leveraging rich, biomedical Semantic Web ontologies to represent medical knowledge, both known and tentative, we increase the accuracy and expressivity of plausible reasoning, and cope with issues such as data heterogeneity, inconsistency and interoperability. In this paper, we present a Semantic Web-based, multi-strategy reasoning approach, which integrates deductive and plausible reasoning and exploits Semantic Web technology to solve complex clinical decision support queries.

**Results:**

We evaluated our system using a real-world medical dataset of patients with hepatitis, from which we randomly removed different percentages of data (5%, 10%, 15%, and 20%) to reflect scenarios with increasing amounts of incomplete medical knowledge. To increase the reliability of the results, we generated 5 independent datasets for each percentage of missing values, which resulted in 20 experimental datasets (in addition to the original dataset). The results show that plausibly inferred knowledge extends the coverage of the knowledge base by, on average, 2%, 7%, 12%, and 16% for datasets with, respectively, 5%, 10%, 15%, and 20% of missing values. This expansion in the KB coverage allowed solving complex disease diagnostic queries that were previously unresolvable, without losing the correctness of the answers. However, compared to deductive reasoning, data-intensive plausible reasoning mechanisms yield a significant performance overhead.

**Conclusions:**

We observed that plausible reasoning approaches, by generating tentative inferences and leveraging domain knowledge of experts, allow us to extend the coverage of medical knowledge bases, resulting in improved clinical decision support. Second, by leveraging OWL ontological knowledge, we are able to increase the expressivity and accuracy of plausible reasoning methods. Third, our approach is applicable to clinical decision support systems for a range of chronic diseases.

**Electronic supplementary material:**

The online version of this article (doi:10.1186/s13040-017-0123-y) contains supplementary material, which is available to authorized users.

## Background

Medical Knowledge-Based Systems (M-KBS) assist physicians in making clinical diagnoses, suggesting therapeutic interventions, recommending treatments, and tutoring other clinicians. To that end, medical KBS perform a variety of functions, including knowledge acquisition, knowledge translation and logic-based reasoning; and utilize data from a range of sources, such as evidence-based medical literature, approved clinical practice guidelines (CPG) and patients’ Electronic Health Records (EHR). Due to this multitude of heterogeneous sources, developing a *complete* and *consistent* clinical knowledge base is challenging. This is exacerbated by the fact that patient data is often incomplete, and much useful knowledge is hidden in tacit physician experiences, e.g., based on perceived interrelations between interventions and outcomes.

Accordingly, solving a clinical case in practice often involves an interplay between objective knowledge, found in published, evidence-based clinical literature; and subjective knowledge, consisting of prior experiences of the physician and diagnostic quality correlations within medical data. Compared to the knowledge engineering cycle, physicians typically make a hypothesis, and then, based on the available objective knowledge (i.e., the Deductive Closure of the KB), they attempt to solve the problem. In case the deductive closure is incomplete with respect to the current clinical case, physicians incorporate their own experiences, empirical evidence and tacit knowledge (i.e., the Plausible Closure of the KB). In this process, the end result is sufficient knowledge to solve the current clinical case-i.e., an Acceptable Deductive Closure of the KB. As a result, the medical decision-making process is often an integration of deductive reasoning and so-called *plausible reasoning*, based on plausible or tentative associations within medical data. We argue that such human plausible reasoning is a useful mechanism to extend the decision-support capabilities of computerized, medical KBS. Although plausible inferences do not guarantee the truth of inferred knowledge, they are a response to reasoning under conditions where there is a lack of complete, explicit knowledge; which is a typical characteristic of physicians’ thought processes.

To cope with other issues such as data heterogeneity, interoperability and inconsistency, Semantic Web technology offer a formal, logic-based framework for modeling the KB. In doing so, the KB can be aligned for interoperation with many existing medical Semantic Web data sources, and Description Logic-based reasoning can be used to detect inconsistencies and infer new facts. To implement rule-based reasoning to deductively infer clinical conclusions, well-known semantic rule languages, such as RuleML [1], SPIN [2] or SWRL [3], can be applied. Finally, semantic KBS provide additional support for plausible reasoning, which is often based on data similarity, by enabling the application of formal, ontology-based conceptual similarity approaches.

### Plausible reasoning

Plausible Reasoning (PR) is a weak inferencing approach, used when a deterministic answer to a question is unavailable. In contrast to classical logic, in which a statement is accepted as truth whenever it is a logical consequence of a complete set of true statements, plausible inference reasons over a partial set of true observations to infer a “likely” true statement [4]. In other words, to cope with missing knowledge, plausible reasoning is conducted on the available evidence and experiments; which are typically empirical, inexact, and uncertain. In this regard, the result of plausible reasoning is not a conclusive answer but rather the best-effort answer in light of what is known so far [5, 6].

Based on findings from argumentation studies on the crossroads of philosophy, reasoning, and logic, Tindale [7] and Walton et al. [4] introduce eleven fundamental characteristics of plausible reasoning. Summarizing these characteristics, plausible reasoning can be recognized as a method which is [8–11]:

- Non-demonstrative: non-demonstrative reasoning depends on knowledge discovery, making hypotheses, and learning new concepts. Typical examples include physician diagnosis, economical statistical evidence, or conclusions of scientific research. On the other hand, demonstrative reasoning (i.e., mathematical proof) is sound, deterministic, beyond controversy, and final, but it is incapable of exploring new knowledge.
- Ampliative: non-ampliative reasoning, like deduction, explicates and instantiates what was already expressed in the captured domain-specific knowledge (e.g., using deductive rules). Instead, ampliative reasoning generates inferences that go beyond what is contained in the captured knowledge.
- Non-monotonic: implies that the validity of a plausible hypothesis can be detracted by a new piece of information.
- Subjective: a plausible argument is an expression of beliefs, opinions, personal preferences, values, feelings, and judgments.

Further, plausible reasoning leverages a set of recurring patterns from human reasoning processes employed to infer answers; which do not necessarily occur in formal logic or non-classical logics such as fuzzy logic [12, 13]. Firstly, plausible reasoning assumes that a large amount of human knowledge is stored in a hierarchical format, which is constantly being updated, modified, and extended. A first category of plausible patterns leverages this hierarchical nature, and includes *generalization*, *specialization*, *similarity*, and *dissimilarity* [14, 15]. A second category of plausible patterns are based on non-hierarchical relations between concepts, namely partial order relations, and includes *interpolation* and *a fortiori*. Table 1 shortly summarizes these patterns.

**Table 1:**
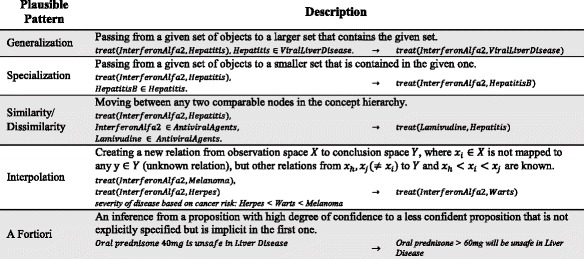
Plausible patterns

Leveraging the relations between concepts and entities, all these patterns manifest new knowledge (ampliative) that is a result of exploring the plausible closure (non-demonstrative) of the KB. Further, these manifestations are non-monotonic, since their validity can be detracted by new, explicit knowledge; and subjective, since their truthfulness strongly depends on the experiences and background knowledge captured in the KB. While these patterns can be used individually, plausible reasoning techniques such as inductive generalization, analogical reasoning, and abduction, are inspired by these patterns to perform more complex reasoning. In this paper, we focus on inductive generalization and analogical reasoning, which we summarize below.

Inductive generalization hypothesizes that similarities between data entities likely account for a particular features in common; this knowledge can then be formalized and generalized in terms of rules. In this regard, *generalization* and *similarity* play a significant role in making an inductive inference [16]. Analogical reasoning is guided by plausible domain knowledge, and implies that in case two data entities are similar in one specific aspect, they are likely similar in another as well [17]. This mechanism, which is guided by a plausible rule, leverages *similarity* and *specialization* patterns. We discuss these approaches in detail in the methods section.

### Semantic medical knowledge bases

The Semantic Web (SW) framework offers a formal, logic-based framework for modeling medical knowledge bases, which allows the re-use of well-established biomedical ontologies for modeling; supports the alignment with the multitude of existing, Semantic Web medical data sources; and allows applying Description Logics (DL)-based reasoning, to check for consistency and infer new clinical facts. Below, we elaborate on the building blocks of Semantic Web technologies, and how they contribute to the representation and reasoning over medical KBS.

The *Resource Description Framework (RDF)* is the underlying data structure of the Semantic Web. RDF is used to make statements about resources (i.e., entities or concepts) in the form of *subject*-*predicate*-*object* triples, allowing both properties of resources as well as relations between resources to be described. In doing so, RDF gives rise to a conceptual graph structure spanning interlinked datasets. Domain-specific ontologies, written using the *Web Ontology Language (OWL)*, introduce the vocabularies required for rich attributional, hierarchical or relational resource descriptions. These ontologies are likewise expressed in RDF and kept together with the instance data, facilitating data exchange. *SPARQL* is a pattern matching-based query language for querying connected RDF graphs, kept in online or offline data sources.

We posit that, due to the availability of DL-based consistency checking and reasoning, together with deductive reasoning based on semantic rule languages, semantic technologies are useful for realizing medical KBS. This observation is reflected by work in KBS also applying Semantic Web technology [18–20]. Further, due to its graph-based nature and support for rich concept hierarchies, we argue that Semantic Web technology provides excellent support for plausible reasoning; which relies on hierarchical and other kinds of entity relations (see previous Section). Further, formal semantic similarity techniques may be applied to more accurately establish entity similarity [18–20].

**Fig. 1:**
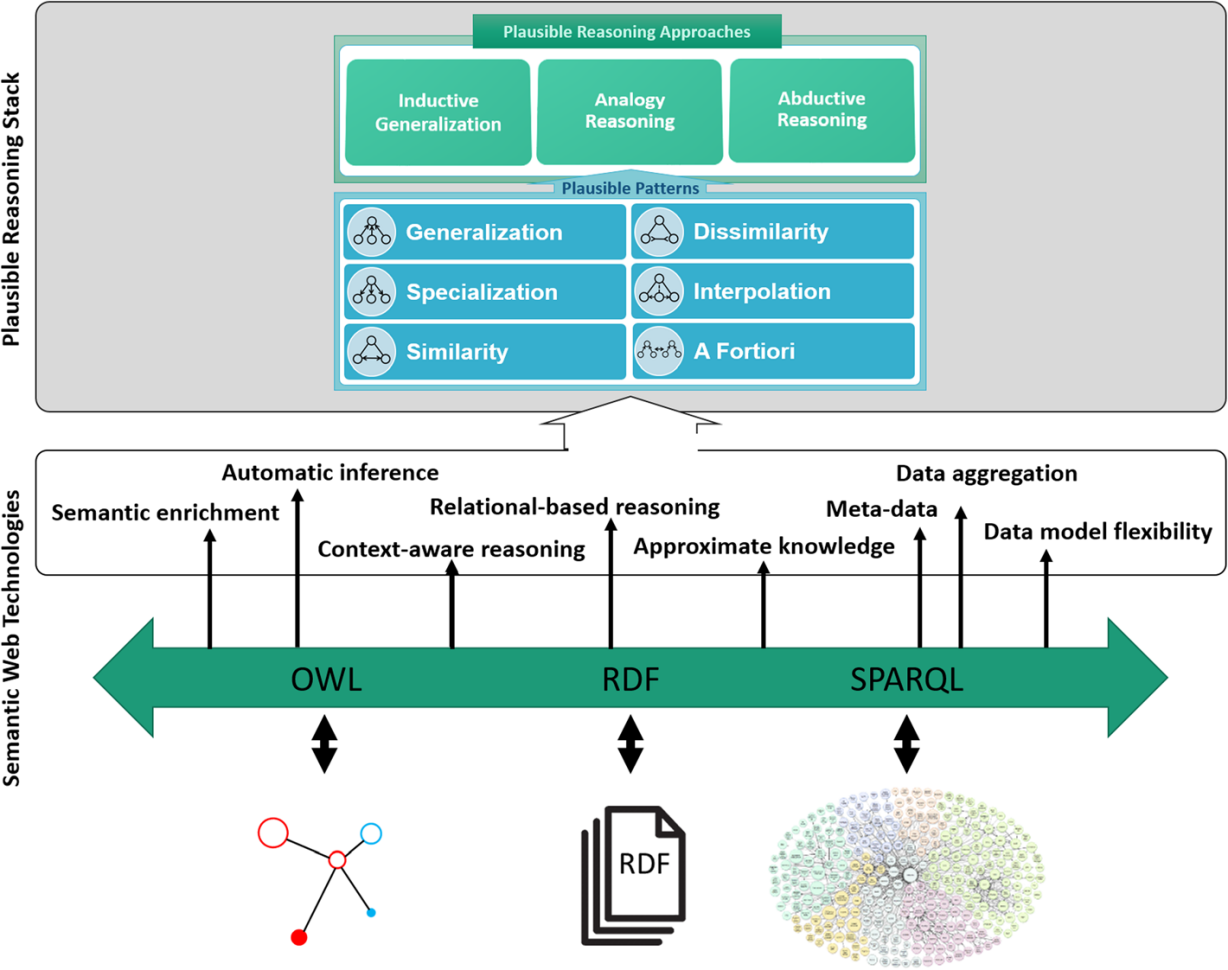
Semantic medical knowledge bases and plausible reasoning – mentioning the three core building blocks of the Semantic Web, namely RDF, OWL, and SPARQL

Figure 1 provides an overview of what Semantic Web technologies offer to medical knowledge bases, and how it can improve the accuracy of weak inferencing methods by providing precise semantics and flexible ways to convey information.

### Uncertainty reasoning in the semantic Web

Although effective, well-established deductive reasoning mechanisms exist, including inferencing based on Description Logics and rule-based languages, there is currently a lack of built-in support for representing and reasoning about uncertainty in Semantic Web technologies. This pitfall not only hampers the Semantic Web in reaching its full potential [21], but also restricts its usage in knowledge-based systems that need to deal with uncertainty and missing knowledge. Studies on dealing with uncertainty within the Semantic Web framework have led to a variety of approaches [22–25], including probabilistic OWL variants, Bayesian Network theories to model probabilistic ontologies and reason with uncertainty, fuzzy extensions to OWL to deal with imprecise and vague information [26–29]. Such probabilistic/fuzzy OWL extensions have shown to improve the capability of Semantic Web reasoners in dealing with uncertainty. However, they are only applicable to cases where the correctness of propositions has some specified degree of ambiguity, not cases where uncertainty is the due to lack of knowledge-i.e., a case of incomplete knowledge.

Agibetov et al. [30] proposed an evidence-based hypothesis testing method in the biomedical domain to cope with incomplete knowledge, where the researchers extract a causal chain from an ontology and represent it as Directed Acyclic Graph, which guides specialists in decision-making. It should be noted that the proposed method only notifies specialists what knowledge is missing for the hypothesis to hold, and does not attempt to generate any new knowledge (based on the available knowledge). We argue that it is useful to explore methods that can result in the generation of new knowledge that potentially supports reasoning (for query answering, decision support, etc.) in a Semantic Web framework. In this regard, we argue that plausible reasoning, implemented within a Semantic Web framework, provides an interesting approach to generate new knowledge/assertions that can be utilized by Semantic Web reasoners.

## Method

In our work, we explore semantics-enabled knowledge engineering approaches to extend the decision-support capabilities of medical KBS, when faced with insufficient knowledge. In particular, we have developed and applied a *multi-strategy* reasoning approach that integrates plausible reasoning methods with deductive reasoning methods to potentially extend the coverage of a KB. Our multi-strategy reasoning approach has two steps:

- Step 1: This step aims to extend the coverage of the existing KB by employing plausible reasoning mechanisms to supplement deductive reasoning in case of missing knowledge and derive new facts/assertions. The newly inferred knowledge is validated by a domain expert and the validated (meaningful, relevant and correct) knowledge is asserted into the KB, thus extending the knowledge coverage of the original KB.
- Step 2: This step involves generating reasoning-based solutions using the extended KB. We use traditional deductive and explanation-based reasoning methods to derive solutions (with logical justifications) to queries posed by the user.

We also argue that the use of Semantic Web based knowledge representation can improve the accuracy of weak inferencing methods, such as plausible reasoning; by improving conceptual similarity checking and enhancing the expressive power of plausible knowledge.

We present SeMS-KBS, a Semantic web medical Knowledge-Based System that implements our *Multi-Strategy* reasoning approach to generate plausible justifications, using plausible and deductive inferencing methods, in response to hypothesis testing or recommendation-seeking queries posed by medical practitioners. The *multi-strategy* reasoning approach aims to (i) infer new knowledge and operate with only partial knowledge using plausible reasoning; (ii) improve the quality of results using Semantic Web technologies; and (iii) supplement Semantic Web technology with plausible (non-demonstrative) reasoning as an additional reasoning method when confronted with incomplete knowledge.

### System architecture

**Fig. 2:**
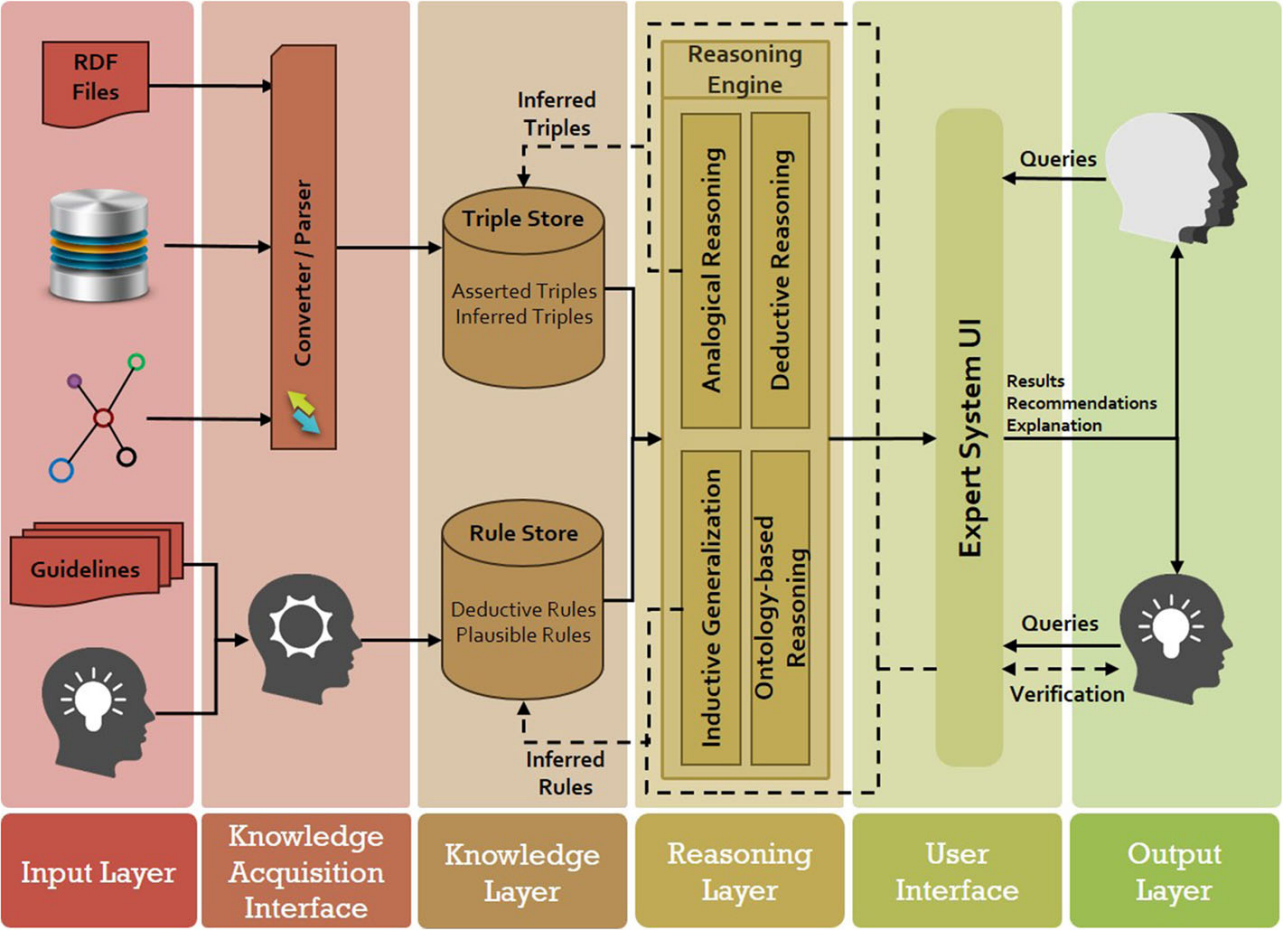
SeMS-KBS process diagram

We have developed a multi-strategy reasoning approach based on Semantic Web technology, which applies deductive and plausible inferencing methods to generate justifications in response to queries posed by medical experts. Figure 2 depicts the architecture of our proposed Semantic Web-based Multi-Strategy KBS (SeMS-KBS). Below, we discuss the different stages in the knowledge base lifecycle, and how they leverage the architecture components. Then, we discuss our multi-strategy reasoning approach, which is the focus of this paper.

#### Stage 1, Knowledge acquisition phase

The *Input Layer* allows the knowledge engineer to gather explicit knowledge from a multitude of sources. These sources include patient data, possibly in different formats and databases, and existing medical knowledge (available texts, journals, and clinical guidelines). Moreover, there is provision for acquiring and representing the tacit medical knowledge and experiences of domain experts [31]. In the *Knowledge Acquisition Interface*, the collected knowledge is formalized in terms of Semantic Web representation, including RDF triples, OWL ontologies, and Semantic Web (SPIN [32]) rules.

#### Stage 2, Knowledge storage phase

The *Knowledge Layer* stores the available knowledge into two different repositories, namely the Triple Store and Rule Store. From a design perspective, separating data and deductive rules simplifies the management of domain knowledge. Both will later be extended with the inferred plausible knowledge from the *Reasoning Phase*. The triple and rule stores are initialized with the (converted) information from the *Knowledge Acquisition Phase*, called *Asserted Triples / Rules*. This part would not change, unless the knowledge engineer makes corrections based on new information. In contrast, the part called *Inferred Triples / Rules* will be supplemented gradually with inferred facts and rules from plausible inferences. As discussed in the *Query-Answering and Justification Phase*, such newly inferred information will only be inserted into the knowledge base after the expert’s investigation and verification.

#### Stage 3, Reasoning phase

The *Reasoning Layer* features a multi-strategy reasoning approach to deal with the initial incompleteness of the KB. This strategy applies the following reasoning methods: (a) Deductive Reasoning, based on deductive rules within the KB; (b) Ontology-based Reasoning, which leverages semantic knowledge from domain-specific ontologies and directly materializes inferences in the KB; (c) Inductive Generalization, making hypotheses based on the commonalities between data entities and generalizing these hypotheses as rules [33]; and (d) Analogical Reasoning, which adds new attributes to a target entity based on its similarity to another entity, as indicated by plausible rules [34].

#### Stage 4, Query-answering and justification phase

In SeMS-KBS, medical experts pose queries via the *User Interface Layer*. The system then attempts to resolve these queries, by triggering the *Reasoning Phase* to generate (plausible) justifications for each query. Further, it provides interfaces for the medical expert to verify any plausible inferences used by the justification, and also confirm particular justifications to add them to the KB permanently. By relying on the medical expert’s tacit knowledge to incorporate (plausible) justifications, we allow their medical experience and best judgement to be reflected in the KB.

### Multi-strategy reasoning approach

SeMS-KBS employs a stepwise approach for explaining or justifying a query posed by medical experts. At its core, the reasoning engine relies on deductive rules to justify why a given query instance could be correct. This step is implemented via backward chaining, which recursively finds deductive rules and knowledge base facts proving the query statement. If this step fails to generate a complete justification (e.g., due to missing knowledge), the system attempts to supplement the justification with plausible inferences, using inductive generalization and analogical reasoning. In any case, a logical first step to supplementing an incomplete, Semantic Web KBS is to apply ontology inferencing; i.e., leveraging domain-specific, ontological knowledge to infer new information.

**Fig. 3:**
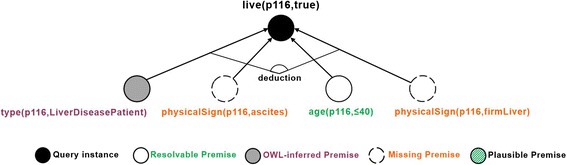
Initial justification tree of the query live(p116,true)

To elaborate our multi-strategy medical reasoning approach, we present a medical case study based on the hepatitis dataset introduced later (Reference Data for Evaluation), whereby SeMS-KBS is queried about the survival of patients with hepatitis. In our case study, we are using the first dataset of the series with 10% missing values (Dataset #10–1) to query a patient called *p116*. Figure 3 visualizes the initial query justification as a tree structure. We note that, for clarity, rules use predicate logic notation (namespaces are omitted), and type restrictions on arguments are abbreviated as nested “(type X)” statements. In this tree, the root represents the posed query; blank leaves with solid lines denote initial facts in the KB; facts inferred via ontology-based reasoning are manifested as grey-shaded leaves; and blank leaves with dashed lines represent missing premises (i.e., not found as facts and unproven by other deductive rules).

Below, we show the deductive rule used by the backward chaining process to build this justification tree:
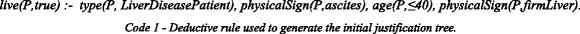


In the initial justification, the premise about the patient’s age was proven by a KB fact. Code 2 shows a snippet of Dataset #10–1 that represents data recorded for patient *p116*:
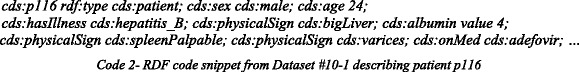


This leaves three premises currently unproven in the justification tree: i.e., whether *p116* is a patient with liver disease, and whether they have physical signs of ascites and a firm liver. Below, we elaborate on how reasoning strategies employed by SeMS-KBS may resolve these missing premises.

### Ontology-based reasoning

The *LiverDiseasePatient* OWL class description defines the set of liver disease patients, and indicates that this set is a subset of all patients (*subClassOf Patient*), and includes all individuals that have a recorded liver disease (*someValuesFrom LiverDisease*). We show this class description below:
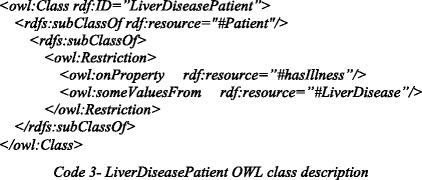


Code 2 shows that *p116* currently has *hepatitis_B*, which is an instance of *Hepatitis*, a subclass of *LiverDisease*. Therefore, SeMS-KBS’s OWL reasoner infers that *p116* is a *LiverDiseasePatient*, fulfilling the first missing premise.

### Analogical reasoning

Analogical reasoning, which is guided by plausible rules, implies that if two entities are similar in a particular aspect, they are plausibly similar in another specific aspect as well [16]. This allows transferring knowledge from a well-known entity to a lesser known, similar entity [17, 35, 36]. We exemplify a plausible rule below, which states that “Q is plausibly determined by P”; i.e., if two entities are both characterized by the same feature P, then they will likely also share feature Q:
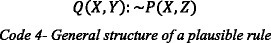


In SeMS-KBS, analogical reasoning is triggered by an incomplete justification tree, and starts by searching the KB for plausible rules that may infer the missing premise. In our medical case study, the domain expert provided a set of plausible rules, including a rule implying that if two entities have the same vein issue (i.e., similar in one aspect), they plausibly have the same liver issue (i.e., similar in another aspect):
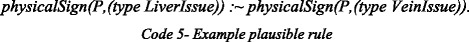


In this plausible rule, ontological knowledge is leveraged to allow arbitrary levels of granularity, thus increasing its expressivity: any kind of vein issue (e.g., varices) suffices as knowledge-transfer condition, and any kind of liver issue (e.g., firm liver) may be transferred to the lesser known entity. Further, we note that only the high-level condition and consequence of the knowledge transferal is specified; the particulars are left up to the concrete entities, as illustrated below.

**Fig. 4:**
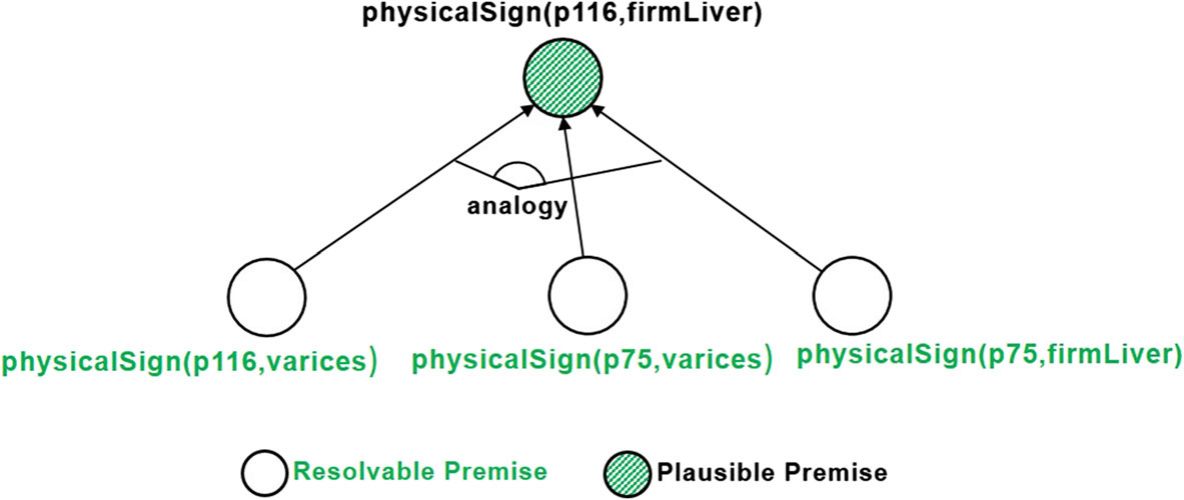
Part of justification tree extended by analogical reasoning

In our case study, using the plausible rule above, the KB is searched for an entity unifying the rule, i.e., matching both rule condition and consequent. Patient *p75* is found to have a particular vein issue (*varices*) and liver issue (*firmLiver*):
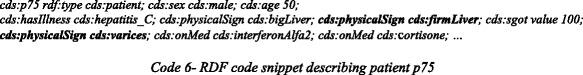


By sharing the same kind of vein issue (*varices*), the plausible rule from Code 5 implies that both patients plausibly share the same liver issue (*firmLiver*) as well, thus allowing us to transfer knowledge from one, well-known entity (*p75*) to a lesser-known one (*p116*). Fig. 4 illustrates the part of the justification tree extended by analogical reasoning.

Pseudocode 1 shows the implementation of analogical reasoning in our knowledge-based system. As mentioned, this process is driven by failed justification premises. In this case, the process starts by looking for plausible rules that can resolve a missing premise (line 1). At this step, the concept hierarchy is leveraged to expand the search space. In this example, the direct superclass of the missing premise *firmLiver* (i.e., *(type LiverIssue)*) will match the consequent of the plausible rule from Code 5. The knowledge base is then searched for facts that *unify* the rule; i.e., match the rule condition and consequent (line 2). For each found fact, the algorithm checks whether its instantiated knowledge-transfer condition matches the original entity (line 3). If so, its consequent is added to the entity, at least in the system’s working memory (line 4). If the expert confirms the overall justification, this working memory will be materialized in the knowledge base (see section *Implementation of SeMS-KBS*).
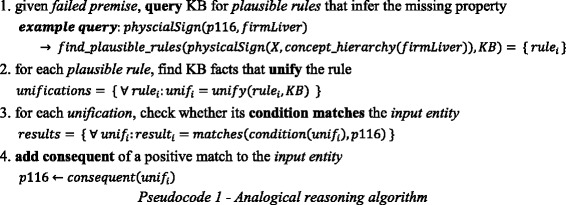


### Inductive generalization

Inductive reasoning generates new plausible knowledge by generalizing similarities between KB entities. Given a particular feature, the knowledge base is searched for a set of entities sharing the feature. Other similarities between these entities are hereby hypothesized as accounting for the feature [16]. Inductive reasoning manifests this hypothesis as the following type of rule:
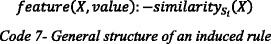


Where *similarity*_*Si*_ (*X*) represents similarities between matching entities *S*_*i*_ (i.e., sharing the particular feature), and *feature*(*X, value*) represents the given feature. By accepting this rule as a deductive one, any future entity that unifies *similarity*_*Si*_ (*X*) will be assumed to exhibit the given feature as well. We note that in this process, a high number of matching entities (*positive coverage*) strengthens the validity of the hypothesis.

As a similarity-based reasoning approach, the validity of a hypothesis in inductive generalization strongly depends on the accuracy of the similarity checking method. Accordingly, an improvement in finding commonalities strengthens entity matching, and consequently, enhances the accuracy of inferred rules. Seeing how our Semantic Web-based KB has expressive OWL ontologies as its disposal, we can apply conceptual similarity checking for this purpose. In our approach, we use conceptual similarity to implement similarity as a plausible pattern.

#### Conceptual similarity

A good basis for finding commonalities is to take the conjunction between entity properties. In case exact matches are lacking, conceptual similarity checking can be used to enhance this process. For example, one patient might use *InterferonAlfa2*, while the other uses *Lamivudine*. Although a simple similarity check would rule these two individuals as different, a conceptual similarity check would infer that both medications are of type *Antiviral*, and thus are conceptually similar to a degree.

Calculating semantic concept similarity/dissimilarity has been studied extensively in the literature (e.g., [37, 38]). Typically, these works determine conceptual similarity by calculating their conceptual distance, indicated by the distance to their closest common subsuming concept. It is further argued that two “specific” concepts, lower in the concept hierarchy, will be more similar than two “abstract” concepts that are higher up [38]. We rely on the well-known measure suggested by Wu and Palmer [37], which considers both conceptual distance and concept specificity:

$$ ConSim\left({C}_1,{C}_2\right)=\frac{2\times {N}_3}{N_1+{N}_2+2\times {N}_3} $$

Formula 1 General structure of an induced rule

**Fig. 5:**
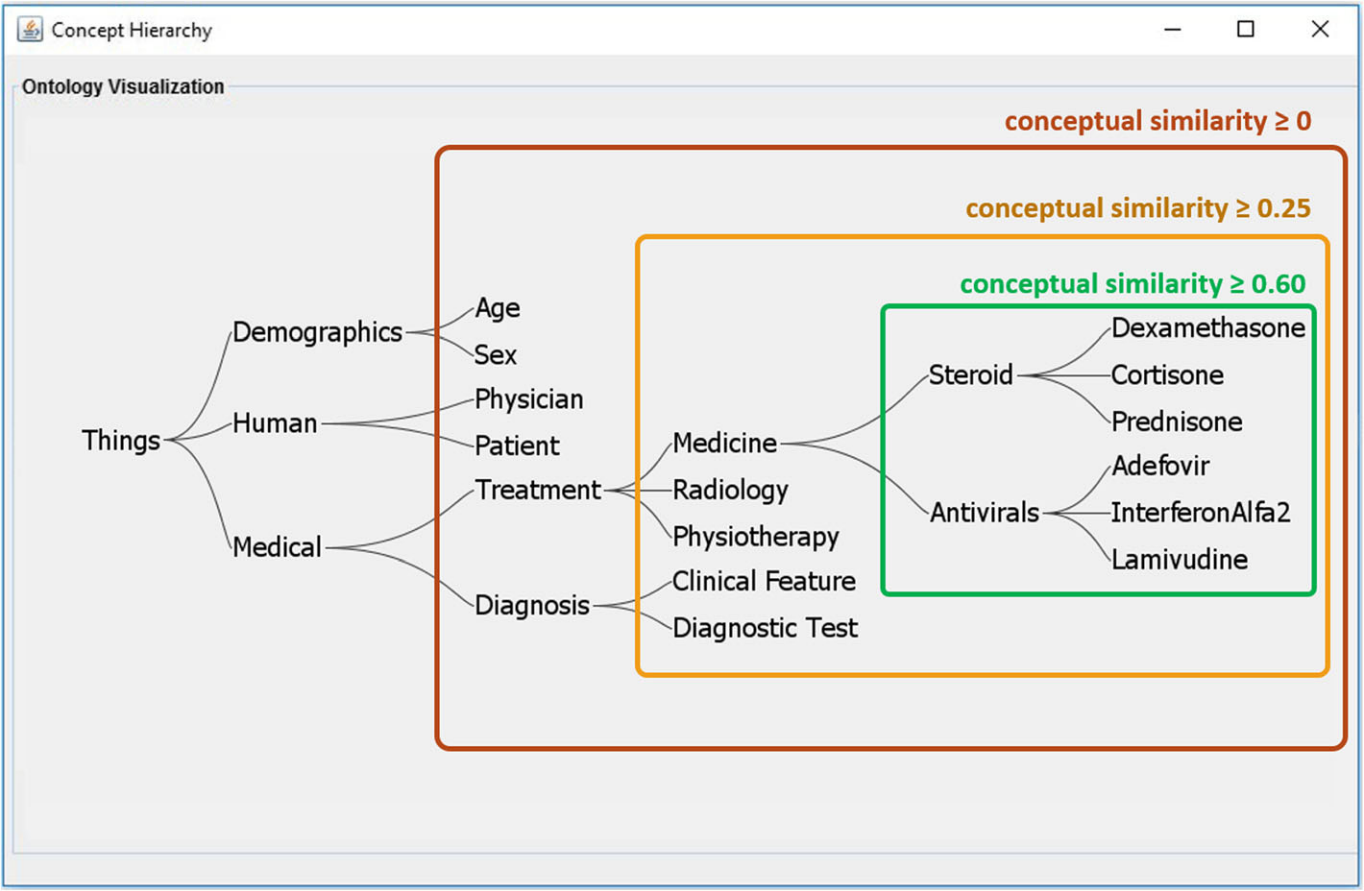
Concept hierarchy of the hepatitis data with corresponding conceptual similarity values-presenting the similarity of clinicial medicines to diagnosis and demographic infomation

By considering C3 as the closest common parent of C1 and C2; then N1 is the number of nodes on the path from C1 to C3, and N2 is the number of nodes between C2 and C3 (indicating conceptual similarity); and N3 is the number of nodes on the path from C3 to the root (indicating conceptual specificity). Using Formula 1, Fig. 5 depicts the similarity between some classes and individual of the ontology.

**Fig. 6:**
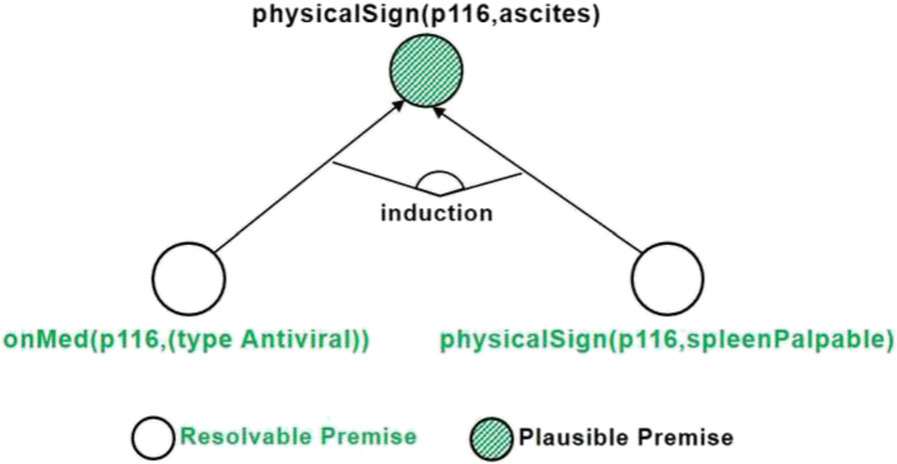
Part of justification tree extended by inductive generalization

Regarding our medical case study, the remaining missing premise is *physicalSign(p116,ascites)*. In the first step, the KB is searched for patients who have this physical sign. The RDF code snippet below describes a subset of patients who share a number of characteristics, as well as the currently missing feature:
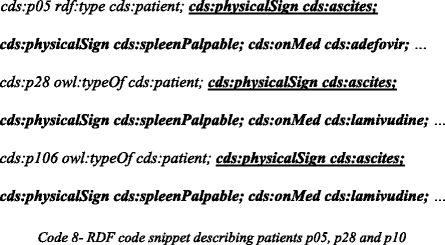


When considering other similarities between these patients, and applying conceptual similarity in the process, we find that they all have a palpable spleen and use medications of type *Antiviral*. The conjunction of these matching properties is taken, resulting in a generalized rule with the missing premise as its consequent and the common properties as its conditions:
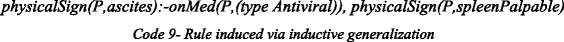


Figure 6 demonstrates the part of the justification tree extended by induction.

Pseudocode 2 shows the pseudocode for inductive reasoning in our system. As before (see Pseudocode 1), the process is triggered by a failed premise in the justification tree. Firstly, the knowledge base is queried with a generalized version of the failed premise, to retrieve all entities with the missing property value (line 1). Subsequently, similarity is determined between matching entities and the query entity (line 2) using the *similarity* function (see Fig. 9). The next step is to aggregate equivalent similarities (i.e., involving the same property values), thus collecting the necessary information (e.g., positive coverage) for each potential deductive rule (line 3). Afterwards, deductive rules are generated based on these aggregated similarities (line 4). These will be presented to the domain expert (see Section 4), who can then choose one of the rules to be added to the knowledge base.
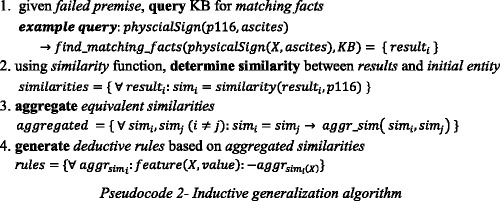


### Challenges of plausible reasoning in SeMS-KBS

It should be noted that plausible knowledge is inferred by plausible reasoning (i.e., inductive generalization or analogical reasoning) methods that are applied to domain-specific data. Like any data analytics/mining strategy, findings of a data-driven model need to be validated by a domain expert for correctness and relevance. Likewise, the knowledge inferred by plausible reasoning needs to be first validated by a domain expert and then it can be augmented to a KB in order to extend its coverage.

Inductive generalization works on establishing correlations between data attributes, hence it is important to filter out attributes that are irrelevant to the query, and thus prevent SeMS-KBS from generating large amounts of invalid rules. This is akin to data preparation and filtering performed in a data mining or KDD process. In our experiments, based on expert advice, we filtered out the *Sex* attribute from the inductive reasoning step since there is no correlation between the gender of a patient and hepatitis disease. In doing so, we avoid burdening the domain expert with large numbers of incorrect induced rules. This data preparation step would need to be repeated for other kinds of chronic illnesses, based on the clinical relevance of data attributes.

## Results and discussion

### Implementation of SeMS-KBS

In SeMS-KBS, the SWI-Prolog engine [39] is used to perform deductive reasoning and the unification steps in the analogical reasoning; Aleph [40], an Inductive Logic Programming (ILP) system, conducts inductive reasoning; and ontology-based inferencing is performed by the Apache Jena [41] OWL reasoner. Custom Java code implements similarity checking, as well as data conversion from RDF triples and SPIN rules to Prolog format and vice versa. In order to connect Java to Prolog, we used JPL 2.0.2 [42]. JPL is a set of Java classes and C functions that provide an interface for Java to connect to a Prolog engine. The Prefuse toolkit [43] is used to visualize justification trees and the ontology hierarchy.

### Working of SeMS-KBS

The working of SeMS-KBS is such that a physician (healthcare provider) queries the system to seek assistance for performing clinical diagnoses or recommending treatments. In the previous section, we elaborated on how the multi-strategy reasoning mechanism incorporates deductive and plausible reasoning in generating query justifications.

**Fig. 7:**
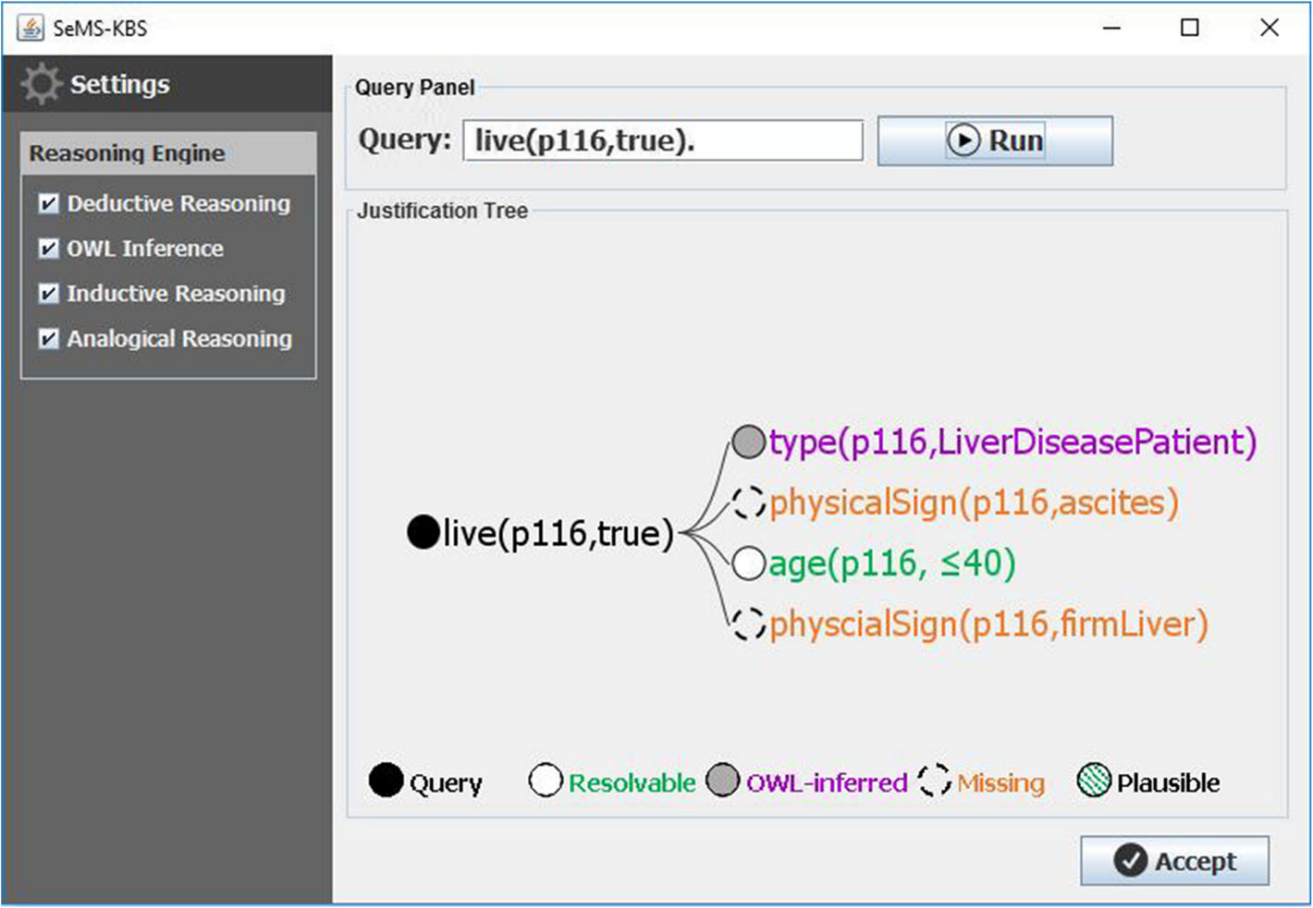
SeMS-KBS User Interface: Main query interface

Below, we illustrate how SeMS-KBS interacts with the user by presenting solutions and their justifications. Figure 7 illustrates the main query interface, which explains the posed query by providing a (plausible) justification tree to the user. Upon clicking a failed premise, plausible resolutions, if any, are shown to the expert for validation.

**Fig. 8:**
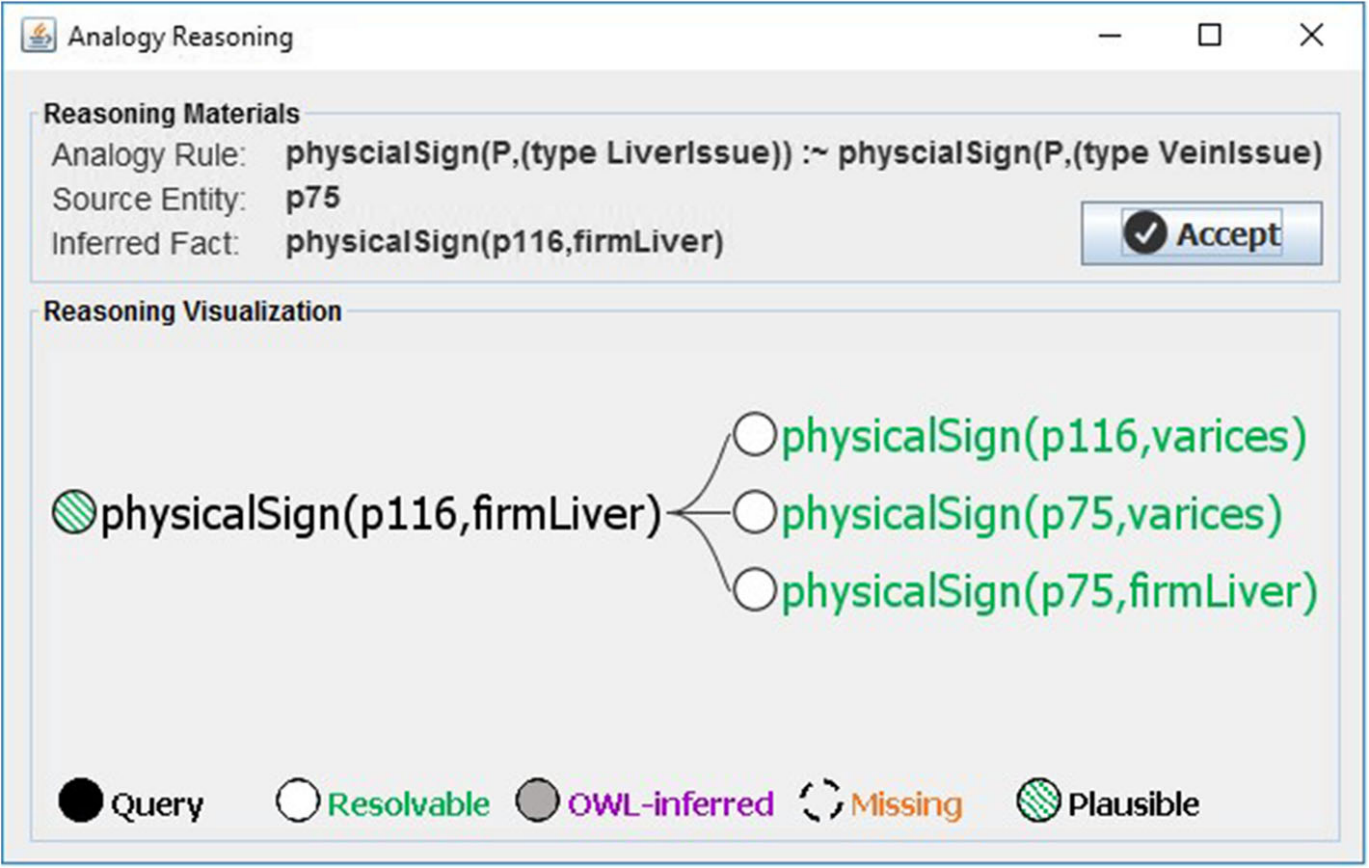
Justification tree generated by analogical reasoning

Figure 8 shows the plausible inference generated by analogical reasoning. The top part provides the reasoning materials used during analogical reasoning, including the analogy rule being applied, the reference entity that unifies the rule, and the inferred fact. The bottom section shows the plausible extension to the justification tree, visualizing the knowledge base facts involved in the inference.

**Fig. 9:**
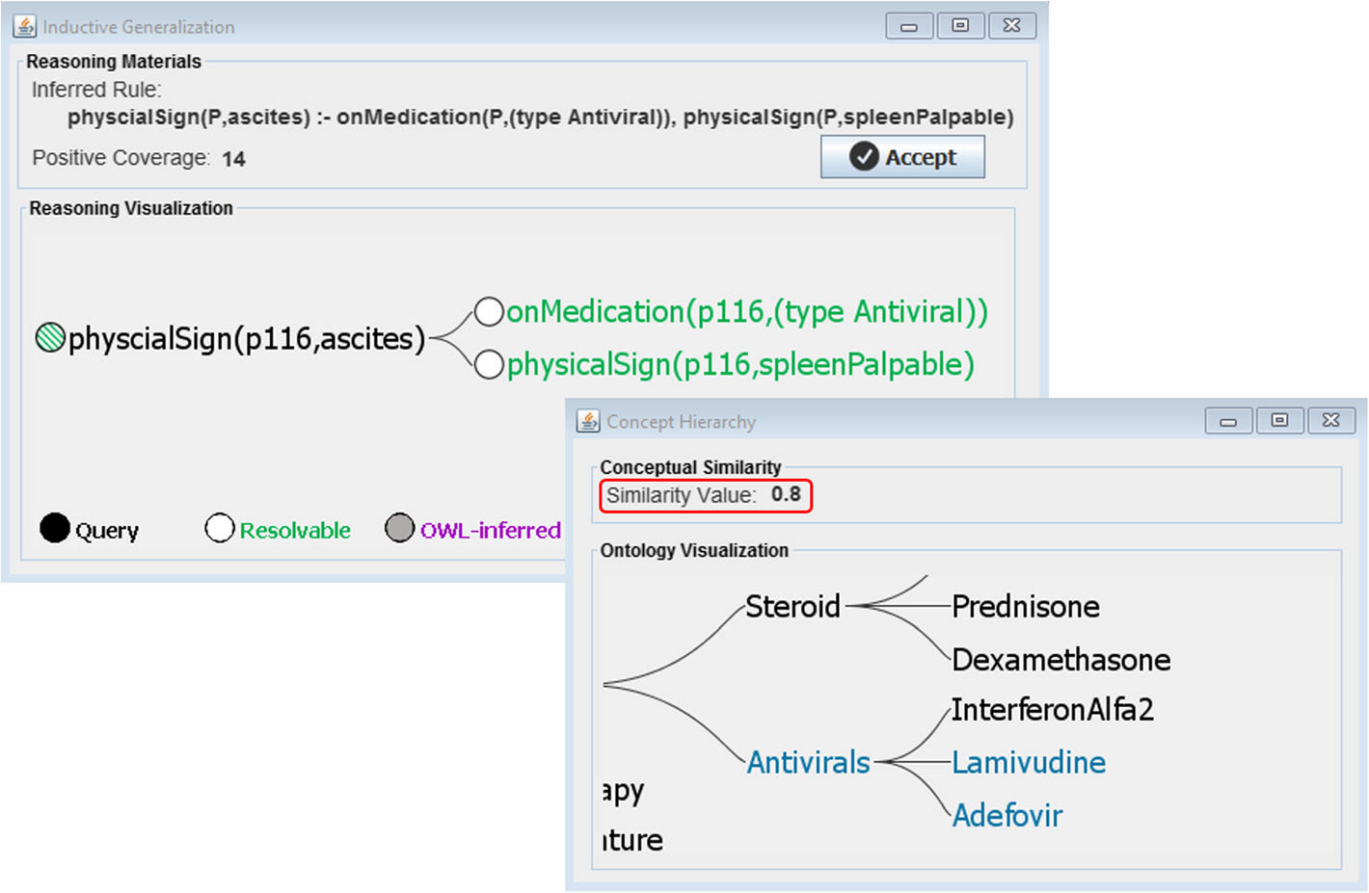
Justification tree generated by induction, and a view of ontology representing the location of Antivirals in the concept hierarchy and the value of conceptual similarity (0.8) between Antivirals and ascites

Figure 9 shows the inductive generalization screen. At the top section, the inferred rule and its positive coverage are provided. Below, the plausible extension to the justification tree is again visualized. A pop-up window is provided to portray the concept hierarchy and indicates the conceptual similarity utilized in the rule induction process.

After the verification of the plausibly inferred knowledge by the expert, this knowledge will be added to the system’s working memory and the justification tree will be updated with the new knowledge. Afterwards, once the domain expert accepts the extended plausible justification, the inferences will be materialized in the knowledge base.

### Reference data for evaluation

To demonstrate the working of our *multi-strategy* reasoning approach, we used a previously published hepatitis dataset [44], retrieved from the UCI Machine Learning Repository [45]. This labeled data describes 155 patients with hepatitis, and includes 20 attributes for each patient. Table 2 summarizes these attributes and their description.

**Table 2:** Summary of reference data - attribute information and distribution

Attribute		Values/Distribution	Description
1	Age	[7 … 78]^a^	Age of the patient
2	Sex	male(139)^b^, female(16)	Gender of the patient
3	Steroids	pos^c^ (78), neg (76)	A class of medication used to provide relief for inflamed areas of the body
4	Antivirals	pos (131), neg (24)	A class of medication used specifically for treating viral infections
5	Fatigue	pos (54), neg (100)	Extreme tiredness, typically resulting from mental or physical illness
6	Malaise	pos (93), neg (61)	A general feeling of discomfort, illness, or uneasiness
7	Anorexia	pos (122), neg (32)	Eating disorder causing people to obsess about weight and what they eat
8	Big Liver	pos (120), neg (25)	Liver is swollen beyond its normal size
9	Firm Liver	pos (84), neg (60)	Liver tissue is harder than normal
10	Spleen Palpable	pos (120), neg (30)	Spleen becomes touchable or bigger than normal size
11	Spiders	pos (99), neg (51)	Small angiomata which appear on the surface of the skin
12	Ascites	pos (130), neg (20)	Accumulation of fluid in the peritoneal cavity, causing abdominal swelling
13	Varices	pos (132), neg (18)	Abnormal veins in lower part of the tube running from throat to stomach
14	Bilirubin	[0.3 … 8.0]	A yellowish pigment found in bile, a fluid made by the liver
15	Alkaline Phosphate	[26 … 295]	A protein found in all body tissues, including the liver, bile ducts, and bone
16	SGOT	[14 … 648]	One of the enzymes that helps liver build and break down proteins
17	Albumin	[2.1 … 6.4]	The main protein of human blood plasma
18	ProTime INR	[0 … 100]	The test that is used to determine the clotting tendency of blood
19	Histology	pos (70), neg (85)	The study of the microscopic anatomy of cells and tissues
20	Patient Label	die (32), live (123)	Entity label, required for evaluating machine learning systems

^a^range of attributes with continuous values

^b^distribution of attributes with categorical values

^c^pos: positive, neg: negative

The numeric ranges show the intervals for continuous values

For categorical values, the numbers of patients with/without that attribute are shown

These attributes can be classified in main six categories as follows:

- *Demographic information*: age, sex
- *Medications*: steroids, antivirals
- *Symptoms*: fatigue, malaise, anorexia
- *Physical signs*: big liver, firm liver, spleen palpable, spiders, ascites, varices
- *Lab tests*: bilirubin, alkaline phosphate, SGOT, albumin, ProTime INR,
- *Pathology*: histology

These categories and their application in the ontology construction step is explained in Data Enrichment section.

### Data preparation

To evaluate the effectiveness of SeMS-KBS when dealing with incomplete data we curated the evaluation dataset in two ways: (i) generation of incomplete datasets with different degrees of incomplete data; and (ii) enriching data with semantic annotations.

#### Generation of test datasets with missing data

The original data already had missing data (167 attribute values). We randomly created incomplete test datasets with 5, 10, 15, and 20% of missing data (compared to the original dataset) to evaluate the performance of SeMS-KBS with increasing amounts of missing information. Using a cross-validation approach, for each missing proportion of a dataset we generated 5 independent datasets-e.g., for the 15% missing dataset there are 5 different datasets each with 15% missing data that is randomly removed from the original dataset. This results in a total of 4 × 5 = 20 randomly generated test datasets.

The generation of the incomplete datasets was performed based on Missing Completely At Random (MCAR) mechanism, in which the missing data is independent of both observed and unobserved data, and missing data are a completely random subset of the initial dataset [46]. Using the Java Math library function, *Math.random,* 1 we calculated a floating-point value between 0 and 1 for each individual fact; in case the random value exceeds the threshold (i.e., 0.9 for datasets with 10% missing values or 0.85 for datasets with 15% missing values), it is left out of the knowledge base.

While the initial KB includes 2778 individual facts about 155 patients, after these data manipulations, Table 3 illustrates the average distribution of missing attribute values among test datasets with different percentage of missing values. To get an insight into how the datasets look like, Additional file 1 and Additional file 2, respectively, show Dataset #10–1 with 10% missing data, and Dataset #20–2 with 20% missing data. Additional file 3, provides the details of missing values for each dataset separately.

**Table 3:** Average number of missing values among test datasets with same percentage of missing values

	Original Dataset	Datasets w. 5% Missing Data	Datasets w.10% Missing Data	Datasets w.15% Missing Data	Datasets w.20% Missing Data
Total # missing values (average)	167	306	438	577	720

#### Data enrichment

The original hepatitis dataset is represented in a raw sequence of data samples, which does not provide any insight of the concepts, their relations, and background knowledge. Although many machine learning algorithms and data mining approaches perform analysis on raw data, Semantic Web reasoners and plausible reasoning approaches require an ontology-based representation, which better reflects the meaning of data along with their interrelationships.

Therefore, there is a need to enrich raw data with semantic information. To this aim, we require integrating different levels of domain knowledge to extend the definition of concepts used in the hepatitis dataset. Attributes of raw data and domain knowledge are the input of our enrichment phase that are converted to an expressive ontology with aim of a domain expert.

To develop the ontology, we used the original attribute types of the dataset as the middle level. By subsequently grouping these attributes into general concepts, we add common super-classes for the attributes. Regarding specialization, we enumerated specific types of medicines for Steroids and Antivirals (see Table 2), two classes of medicine that are often prescribed to hepatitis patients. In particular, we listed each medication type with the following instances:

- *Steroid*: Cortisone, Prednisone, Dexamethasone
- *Antiviral*: InterferonAlfa2, Lamivudine, Adefovir

**Fig. 10:**
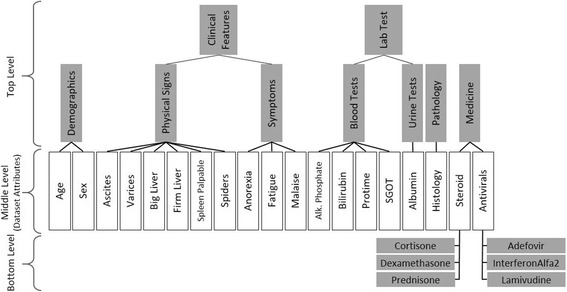
A partial view of the developed ontology after the data enrichment phase

The output of the data enrichment phase is an ontology which clearly reflects the relations between the attributes and allows the system to perform ontology-based reasoning. Figure 10 depicts part of this ontology (the complete ontology is provided in Additional file 4).

### Construction of the knowledge base

The reference data described above is used as the contents of the knowledge base. To equip SeMS-KBS with the required (plausible) domain knowledge necessary for reasoning, a domain expert (in this case, a physician) provided deductive and plausible rules (explained in section Analogical Reasoning), resulting in 9 deductive and 7 plausible rules, which are used in all the experiments. To support ontology-based reasoning, based on the hepatitis dataset, we developed an ontology (see Fig. 10, and Additional file 4) for our application.

### Evaluation

Different studies [47, 48] introduce different criteria for the evaluation of a KBS. Important criteria hereby include completeness, reliability, effectiveness and usefulness of responses, transparency and explanation adequacy. In our work, we investigate the efficiency and performance of the SeMS-KBS two metrics: (i) Coverage and (ii) Correctness. Coverage represents the ratio of the number of answered queries (either correct or incorrect) to the total number of queries (which is 155 in our case study), while correctness shows which percentage of those answered queries were answered correctly (i.e., the ratio of correctly answered queries to queries that were answered either correct or incorrect). The below formulas illustrate the calculation formula of coverage and correctness.

- $$ Coverage = \frac{Total\  No.\kern0.5em  of\  Patients- No.\  of\  Unresolved\  Queries\ }{Total\  No.\kern0.5em  of\  Patients} $$
- $$ Correctness = \frac{Total\  No.\kern0.5em  of\  Patients- No.\  of\  Unresolved\  Queries - No.\  of\  Incorrect\  Answers}{Total\  No.\kern0.5em  of\  Patients - No.\  of\  Unresolved\  Queries} $$

Formula 2 Principle of calculating coverage and correctness ratios

### Design of the experiments

In the experiments, SeMS-KBS is loaded with a hepatitis dataset (discussed in Reference Data) to evaluate the efficiency of SeMS-KBS in dealing with different degrees of missing data; the original dataset, which contains a small number of missing data, and datasets with different percentages (5%, 10, 15%, and 20%) of missing data. For each dataset, a medical expert (in our case, a physician) asked the system to justify the query *live(P,true)*, with the goal of diagnosing the possibility of survival for each patient. For each incomplete justification (i.e., for which the system has no answer), inductive and analogical reasoning are applied to supplement deductive reasoning with plausible inferences. Using their best judgement and medical experience, the medical expert then confirmed or refuted these (plausible) justifications.

Results below demonstrate the proficiency of the system in justifying the non-resolvable queries and determine the relationship between the amount of missing values and the plausible extension to the KB.

### Experimental results

Table 4 presents the result of SeMS-KBS with the original dataset. With 167 missing values, deductive reasoning can provide answers for 151 patients, including 144 correct and 7 incorrect answers (based on the labels of the patients in the original dataset), and leaves 4 patients unresolved. After supplementing deductive reasoning with all plausibly inferred knowledge (without expert’s verification), only 1 patient remains unresolved and 5 answers are incorrect. The results also show that removing inapplicable inductive rules (by expert’s determination) increases the number of wrong answers (6 patients) and unresolved queries (2 queries). To explain this discrepancy, we note that expert’s verification may involve refuting justifications that yield a correct answer, but are nevertheless incorrect since their constituent plausible inferences are not acceptable from a clinical standpoint (e.g., inapplicable induced rules; see Section *Challenges of Plausible Reasoning in SeMS-KBS*). Although expert’s verification leads to a slight decrease in the both coverage and correctness, it is still higher than the correctness of deductive reasoning alone, which shows that SeMS-KBS is capable of extending coverage without losing correctness.

**Table 4:** Experimental results of the original dataset

	Reasoning Strategy		
	Deductive Only	Deductive + Plausible (Without Expert's Verification)	Deductive + Plausible (With Expert’s Verification)
#Queries answered correctly	144	149	147
#Queries answered incorrectly	7	5	6
#Queries unanswered	4	1	2
# Plausible rules used	N/A	3	2
# Plausible facts used	N/A	1	1
Coverage	97%	99%	99%
Correctness	95%	97%	96%

In addition, Table 4 provides coverage and correctness ratios, as we found them useful criteria to compare the results of different reasoning strategies, as well as different datasets. Using the ratios above, Table 4 is considered as the ‘gold standard’ that shows the maximum coverage and correctness that can be achieved through different reasoning strategies, using the original dataset as the most complete dataset.

Table 5 shows the results of experiments with higher amounts of missing values, which helps us to investigate 1) the effect of increasing missing knowledge on the coverage and correctness of the answers, 2) the ability of SeMS-KBS to cope with this increase in missing knowledge, and 3) check whether the findings of the first experiment with the original dataset is generalizable to the other datasets.

**Table 5:** Experimental results of the datasets with different percentages of missing data

Reasoning Strategy		Deductive Only	Deductive + Plausible (Without Expert’s Verification)	Deductive + Plausible (With Expert’s Verification)	Deductive Only	Deductive + Plausible (Without Expert’s Verification)	Deductive + Plausible (With Expert’s Verification)	Deductive Only	Deductive + Plausible (Without Expert’s Verification)	Deductive + Plausible (With Expert’s Verification)	Deductive Only	Deductive + Plausible (Without Expert’s Verification)	Deductive + Plausible (With Expert’s Verification)	Deductive Only	Deductive + Plausible (Without Expert’s Verification)	Deductive + Plausible (With Expert’s Verification)
		Dataset #5–1			Dataset #5–2			Dataset #5–3			Dataset #5–4			Dataset #5–5		
5% Missing Data	#Queries answered correctly	141	150	144	136	148	138	139	150	144	140	150	145	142	149	144
	#Queries answered incorrectly	7	4	7	8	6	8	8	4	9	8	4	8	7	3	7
	#Queries unanswered	7	1	4	11	1	9	8	1	2	7	1	2	6	3	4
	# Plausible Rules	N/A	3	2	N/A	5	1	N/A	6	3	N/A	2	2	N/A	3	1
	# Plausible Facts	N/A	4	1	N/A	1	4	N/A	3	3	N/A	2	2	N/A	1	2
	Coverage	95%	99%	97%	93%	99%	94%	95%	99%	99%	95%	99%	99%	96%	98%	97%
	Correctness	95%	97%	95%	94%	96%	95%	95%	97%	94%	95%	97%	95%	95%	98%	95%
		Dataset #10–1			Dataset #10–2			Dataset #10–3			Dataset #10–4			Dataset #10–5		
10% Missing Data	#Queries answered correctly	128	155	144	135	150	142	133	151	144	131	152	141	137	151	143
	#Queries answered incorrectly	8	0	8	6	3	8	7	3	7	10	3	8	6	3	6
	#Queries unanswered	19	0	3	14	2	5	15	1	4	14	0	6	12	1	6
	# Plausible Rules	N/A	4	3	N/A	4	1	N/A	7	3	N/A	4	2	N/A	6	2
	# Plausible Facts	N/A	3	4	N/A	3	2	N/A	1	2	N/A	1	8	N/A	3	3
	Coverage	88%	100%	98%	91%	99%	97%	90%	99%	97%	91%	100%	96%	92%	99%	96%
	Correctness	94%	100%	95%	96%	98%	95%	95%	98%	95%	93%	98%	95%	96%	98%	96%
		Dataset #15–1			Dataset #15–2			Dataset #15–3			Dataset #15–4			Dataset #15–5		
15% Missing Data	#Queries answered correctly	119	150	146	128	151	140	124	147	139	113	147	141	128	150	142
	#Queries answered incorrectly	9	4	7	9	4	10	9	5	9	8	8	9	9	4	8
	#Queries unanswered	27	1	2	18	0	5	22	3	7	34	0	5	18	1	5
	# Plausible Rules	N/A	6	5	N/A	5	2	N/A	5	4	N/A	8	4	N/A	6	3
	# Plausible Facts	N/A	1	1	N/A	3	2	N/A	0	0	N/A	3	3	N/A	1	3
	Coverage	83%	99%	99%	88%	100%	97%	86%	98%	95%	78%	100%	97%	88%	99%	97%
	Correctness	93%	97%	95%	93%	97%	93%	93%	97%	94%	93%	95%	94%	93%	97%	95%
		Dataset #20–1			Dataset #20–2			Dataset #20–3			Dataset #20–4			Dataset #20–5		
20% Missing Data	#Queries answered correctly	111	143	135	115	152	138	113	150	140	111	148	135	113	148	129
	#Queries answered incorrectly	9	10	10	9	3	10	12	5	10	10	7	13	11	7	13
	#Queries unanswered	35	2	10	31	0	7	30	0	5	34	0	7	31	0	13
	# Plausible Rules	N/A	6	5	N/A	8	3	N/A	7	3	N/A	6	3	N/A	8	4
	# Plausible Facts	N/A	4	5	N/A	1	1	N/A	4	4	N/A	3	4	N/A	4	4
	Coverage	77%	99%	94%	80%	100%	95%	81%	100%	97%	78%	100%	95%	80%	100%	92%
	Correctness	93%	93%	93%	93%	98%	93%	90%	97%	93%	92%	95%	91%	91%	95%	91%

Table 5 shows that every additional 5% of missing values reduces the coverage of deductive reasoning by almost 5%, with the initial coverage of 97% being gradually decreased to 79% (on average) in the final datasets. On the other hand, correctness is only reduced by a total of 3%, with 95% in the original dataset to 92% in the final datasets.

Regardless of the amount of missing values, plausible reasoning (with/without expert’s verification) significantly improves the coverage (ca. 99% without verification and 97% with verification) while the correctness remains stable (ca. 96% in experiments without verification and 94% with verification).

As with the first experiment (see Table 4), the results show that expert’s verification does not increase the correctness-since some correctly answered queries were nevertheless generated using inapplicable inductive rules, and were thus ruled out by the expert. Leaving out such rules also reduces the coverage of experiments with expert’s verification vs. the experiments without verification, as some answers are no longer being returned.

Table 6 summarizes the coverage and correctness of SeMS-KBS, when equipped only with deductive reasoning, and when additionally, leveraging plausible inferencing with and without expert verification. This table shows the averages of the results provided in the Table 5. If we limit the analysis of the results to the experiments with expert’s verification, on average, we see 2%, 7%, 12%, and 16% extension in the coverage of datasets with respectively 5%, 10%, 15%, and 20% of missing values, without losing the correctness (compared to *Deductive Only*).

**Table 6:** Summary of the experimental results of original dataset and datasets with different percentages of missing data-values are average of the corresponding values in Table 5

Reasoning Strategy	Deductive Only	Deductive + Plausible (Without Expert’s Verification)	Deductive + Plausible (With Expert’s Verification)	Deductive Only	Deductive + Plausible (Without Expert’s Verification)	Deductive + Plausible (With Expert’s Verification)	Deductive Only	Deductive + Plausible (Without Expert’s Verification)	Deductive + Plausible (With Expert’s Verification)	Deductive Only	Deductive + Plausible (Without Expert’s Verification)	Deductive + Plausible (With Expert’s Verification)	Deductive Only	Deductive + Plausible (Without Expert’s Verification)	Deductive + Plausible (With Expert’s Verification)
	Original Dataset			5% Missing			10% Missing			15% Missing			20% Missing		
#Queries answered correctly (Avg.)	144	149	147	140	149	143	133	152	143	122	149	142	113	148	135
#Queries answered incorrectly (Avg.)	7	5	6	8	4	8	7	2	7	9	5	9	10	6	11
#Queries unanswered (Avg.)	4	1	2	8	1	4	15	1	5	24	1	5	32	0	8
# Plausible Rules (Avg.)	N/A	3	2	N/A	4	2	N/A	5	2	N/A	6	4	N/A	7	4
# Plausible Facts (Avg.)	N/A	1	1	N/A	2	2	N/A	2	4	N/A	2	2	N/A	3	4
Coverage (Avg.)	97%	99%	99%	95%	99%	97%	90%	99%	97%	85%	99%	97%	79%	100%	95%
Correctness (Avg.)	95%	97%	96%	95%	97%	95%	95%	98%	95%	93%	97%	94%	92%	96%	92%

### Inferred knowledge and expert’s verification

To gain insight on how plausibly inferred knowledge (including rules and facts) aids SeMS-KBS to resolve previously unresolvable queries, Table 7 shows all the *correctly* (and verified) inferred rules and facts after plausible reasoning was applied in the experiments with Dataset #10–1 (Additional file 1) and Dataset #20–1 (Additional file 2).

**Table 7:**
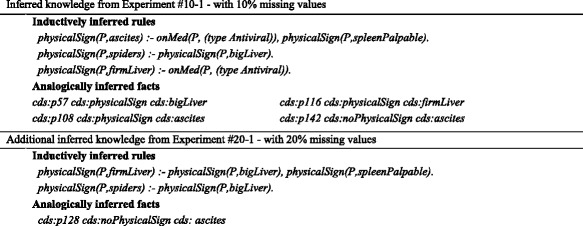
Verified knowledge extension, inferred via induction and analogical reasoning through Experiment #10–1 and #20–1

The results from Experiment #10–1, with 10% missing values, show that the KB is extended with three inductive rules and four facts, which leads to justifying the query *live(P, true)* for an additional 16 out of 19 previously unresolvable patients (Table 5). Experiment #20–1, with 20% missing values, yields two extra inductive rules and one fact (i.e., in addition to the rules and facts from Experiment #10–1). This KB extension justifies the query for 25 patients out of 35 previously unresolvable patients.

As indicated, not all the plausibly inferred knowledge is rationally acceptable to the expert. Table 7 only provides the correct part of the inferred knowledge (i.e., acceptable to the expert), although inductive generalization also generated a number of unrealistic rules, such as:
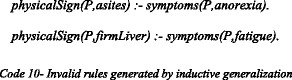


These rules are not medically valid, since the presence of anorexia or fatigue alone does not necessarily imply physical signs of hepatitis (i.e., ascites or firm liver); these rules are only generated due to inductive bias. In our experiment, the expert correctly ruled out these erroneous rules, which in some experiments resulted in a slightly increased correctness (i.e., in Experiment #10–1, correctness is increased to 95 vs. 94% without Expert Verification). At the same time, leaving out such rules causes a decrease in the coverage as some (correct) answers were no longer being returned (Table 5). Table 8 provides an overview of the verified rules that justify the unanswered queries either alone, or with the cooperation of each other, or in the combination with analogically inferred facts. For the sake of simplicity, the inferred rules for datasets with same percentage of missing values are aggregated together.

**Table 8:** Summary of the verified inductively inferred rules, aggregated based on the experiments with same percentage of missing values

Dataset	Verified Inductive Rule	Inferred in more than one Experiment
Original Dataset	noPhysicalSign(P,bigLiver) :- onMed(P, (type Antiviral)).	*
	physicalSign(P,firmLiver) :- symptom(P, malaise).	*
5% Missing	physicalSign(P,ascites) :- physicalSign(P,spleenPalpable).	-
	physicalSign(P,firmLiver) :- onMed(P, (type Antiviral)).	-
	physicalSign(P,firmLiver) :- symptom(P, malaise).	*
	noPhysicalSign(P,spiders) :- noPhysicalSign(P,firmLiver), noPathology(P,histology).	-
10% Missing	noPhysicalSign(P,ascites) :- onMed(P, (type Antiviral)).	*
	physicalSign(P,ascites) :- onMed(P, (type Antiviral)), physicalSign(P,spleenPalpable).	*
	noPhysicalSign(P,bigLiver) :- onMed(P, (type Antiviral)).	*
	noPhysicalSign(P,firmLiver) :- onMed(P, (type Antiviral)), PhysicalSign(P,ascites).	-
	noPhysicalSign(P,firmLiver) :- onMed(P, (type Antiviral)).	-
	physicalSign(P,firmLiver) :- onMed(P, (type Antiviral)).	*
	physicalSign(P,spiders) :- physicalSign(P,ascites).	*
	physicalSign(P,spiders) :- physicalSign(P,bigLiver).	*
	physicalSign(P,spiders) :- physicalSign(P,spleenPalpable).	*
15% Missing	noPhysicalSign(P,ascites) :- onMed(P, (type Antiviral)).	*
	physicalSign(P,ascites) :- physicalSign(P,firmLiver).	-
	physicalSign(P,ascites) :- onMed(P, (type Steroid)).	*
	physicalSign(P,ascites) :- physicalSign(P,varices).	-
	noPhysicalSign(P,bigLiver) :- onMed(P, (type Antiviral)).	*
	physicalSign(P,firmLiver) :- onMed(P, (type Antiviral)).	*
	physicalSign(P,firmLiver) :- physicalSign(P,varices).	*
	physicalSign(P,spiders) :- physicalSign(P,ascites).	*
	physicalSign(P,spiders) :- physicalSign(P,bigLiver).	*
	physicalSign(P,spiders) :- onMed(P, (type Antiviral)).	-
20%Missing	noPhysicalSign(P,ascites) :- onMed(P, (type Antiviral)).	*
	physicalSign(P,ascites) :- onMed(P, (type Antiviral)), physicalSign(P,spleenPalpable).	*
	physicalSign(P,ascites) :- physicalSign(P,firmLiver), physicalSign(P,varices).	-
	physicalSign(P,ascites) :- physicalSign(P,spiders)	-
	physicalSign(P,ascites) :- onMed(P, (type Steroid)).	*
	physicalSign(P,ascites) :- physicalSign(P,varices), physicalSign(P,spleenPalpable).	-
	physicalSign(P,firmLiver) :- onMed(P, (type Antiviral)).	*
	physicalSign(P,firmLiver) :- physicalSign(P,bigLiver), physicalSign(P,spleenPalpable).	-
	physicalSign(P,firmLiver) :- physicalSign(P,spiders).	-
	physicalSign(P,firmLiver) :- physicalSign(P,spleenPalpable).	-
	physicalSign(P,firmLiver) :- physicalSign(P,varices).	*
	physicalSign(P,spiders) :- physicalSign(P,bigLiver).	*
	physicalSign(P,spiders) :- physicalSign(P,ascites),physicalSign(P,varices).	-
	physicalSign(P,spiders) :- physicalSign(P,ascites).	*
	physicalSign(P,spiders) :- physicalSign(P,spleenPalpable).	*

Symbol (*) means yes, and (−) means no

As Table 8 shows, the number of inferred rules grows as the amount of missing values increases. In particular, the greater *diversity* of missing data, which differs per patient, is the main driver behind the increased number of required rules. For example, to infer the missing premise *physicalSign(P,firmLiver)* in experiments with 5% missing values, only two inductive rules suffice, since all the queried patients with missing premise *firmLiver* match either *onMed(P, (type Antiviral))* or *symptom(P, malaise)*. In the experiments with 20% missing values, more rules need to be generated to cover the diversity of missing data for each patient query (e.g., some patients do not have a listed antiviral medication, but instead a variety of physical signs). Consequently, more number of rules with *firmLiver* in the head part, and variety of features in the body part are required.

Table 8 also indicates the consistency of inductive generalization. Although the system was loaded with datasets with different degrees of missing data, most of the inductively inferred rules occur among various experiments-25 rules, out of a total of 40 inferred rules, have re-occurred among all the experiments.

### Speed performance of SeMS-KBS

We further measured the response times of our plausible reasoning mechanisms. Table 9 summarizes the recorded reasoning speeds. In contrast to the trivial response times of analogical reasoning (with an average of 41 ms), the processing time of inductive generalization is dramatically higher (with an average of 10s). This discrepancy can be explained by looking at the details of each methods. Analogical reasoning is guided by plausible rules provided by the expert, while inductive generalization infers a rule by checking similarity among all entities who share a missing feature. This requires a comprehensive search among the KB, resulting in considerable processing time compared to analogical reasoning.

**Table 9:** Speed performance of the SeMS-KBS

Reasoning Method	Minimum	Maximum	Average	STDEV
Inductive Generalization (seconds)	5.5 s	14.5 s	10.3 s	3.8 s
Analogical Reasoning (milliseconds)	6 ms	164 ms	41 ms	40 ms

As mentioned, aside from the performance of the system and usability of its outputs, a KBS should be evaluated from other aspects as well. Transparency and explanation adequacy [48] of a KBS allow users to understand the problem solving and justification procedure of the system. In SeMS-KBS, presenting the supporting materials of justifications and plausible inferences (Figs. 8 and 9) provides a deep insight for the user on generated query explanations and how missing premises are resolved.

A KBS system should also be flexible enough to incorporate changes into the KB during its lifetime. This characteristic is referred to as scalability or incrementalism [49]. An important goal of our system is to let the cooperation between expert and system complement the knowledge base-thus also allowing medical experts to further incorporate their tacit knowledge and experience. Finally, the adaptability [50] of a KBS implies that the deployment of new technologies, methodologies or approaches in a KBS should be conducted with minimal amount of tuning. As the architecture of the SeMS-KBS (Fig. 2) depicts, the modularity and layered structure of the system facilitates the individual modification of different parts of the system, without requiring changes in other parts.

#### Experiment environment

the experiments were performed on a desktop computer with the system configuration (both hardware and software) as follows:

### Hardware

- Operating System: Windows 10 Home (64-bit)
- CPU: Intel® Core™ i7-4770 CPU @ 3.40GHz
- RAM: 12.0 GB Dual-Channel DDR3
- HDD: Seagate 2 TB SATA-III 6.0Gb/s (7200 RPM)
- Graphics: 1023 MB NVIDIA GeForce GTX 645 (NVIDIA)

### Software

- Java JDK 1.7.0
- Java JRE 1.8.0
- Eclipse - Standard Luna-R (win32-×86)
- SWI-Prolog (Multi-threaded, 64 bits, Version 5.10.4)

## Conclusion

This study investigated the potential of implementing plausible reasoning within a Semantic Web framework, in order to extend the coverage of a traditional KB so that it can be used for decision-making and problem solving when confronted with incomplete knowledge. The featured SeMS-KBS specifically targets medical decision-making scenarios, since medical reasoning scenarios often involve gaps in domain knowledge which restrict the generation of deterministic solutions. At the same time, however, there is typically a vast patient data resource that can be leveraged to generate new and missing knowledge, inferred from both the programmed rules as well as domain-specific data.

The main contribution of the paper is demonstrating the utility of plausible reasoning methods for hypothesis testing and decision support. We have shown that traditional deterministic reasoning approaches when augmented with plausible reasoning methods, can provide solutions which would not be possible when working with deductive reasoning methods only. We recognize that plausible reasoning is a weak form of reasoning, but when augmented with external validation by domain experts and application of deterministic reasoning, the solutions provided are reasonably reliable and the justifications can be verified to understand the logic used to derive the solution.

The paper also contributes to the Semantic Web domain by demonstrating the implementation of plausible reasoning within a Semantic Web framework, and this can be an important step to counter uncertainty resulting from missing knowledge. We further showed that the enriched OWL ontologies, detailing semantic relations between concepts, can support concept similarity checking to increase the accuracy of plausible reasoning. By applying inductive generalization, underlying associations between data attributes can be inductively derived-these data-driven inductive generalizations can be treated as new association rules that extend the knowledge coverage of a KB.

Our experimental results confirm that our multi-strategy reasoning approach has good tolerance to missing/incomplete knowledge, and in turn the plausibly inferred knowledge extends the coverage of the KB. The experimental results indicate that the SeMS-KBS is able to extend the KB coverage by 2% (with 5% missing data), 7% (with 10% missing data), 12% (with 15% missing data), and 16% (with 20% missing data). Comparing to deductive only reasoning strategy, validated plausible reasoning results (i) justify, on average, 40%, 70%, 80%, and 75%, respectively, of the queries that were unresolvable, (ii) extend the coverage of the KB to 92–98% (among all the experiments) while never causing the correctness to drop by more than 1%.

Our approach combines machine inferencing capabilities with the tacit, medical experience of human experts, resulting in human-verified knowledge bases. However, in scenarios involving huge amounts of data, it may be impractical to ask a medical expert to manually verify and fine-tune all missing knowledge. In this regard, automatic rule merging [51], to improve the reliability of inductively inferred rules, is considered future work. Additionally, future work involves applying additional plausible patterns (Table 1), and studying how they may be used to supplement plausible justifications. Currently, inductive generalization and analogical reasoning rely on generalization, specialization and similarity/dissimilarity to conduct plausible inferencing. However, the utility of relation-based patterns in making plausible inferences, including *a fortiori* and *interpolation*, has not yet been studied. To this aim, a knowledge base with a variety of semantic annotations and relationships is required. Of course, a larger amount and diversities of data would be helpful since plausible patterns are data-driven methods, guided by hierarchical and relation-based patterns. Therefore, a larger amount of data would strengthen the discovery of hidden relationships among data. Finally, as seen in the performance results, inductive generalization causes a considerable increase in the overhead of the system. Investigating the efficiency of other inductive reasoning approaches/engines, rather than Aleph [40], will be explored in the future work.

## Additional files

Additional file 1: The knowledge base used in Experiment 1 is available at https://goo.gl/5hBCDP. (TXT 71 kb)
Additional file 2: The knowledge base used in Experiment 2 is available at https://goo.gl/hUSV0e. (TXT 62 kb)
Additional file 3: Details of missing values for each test dataset is available at https://goo.gl/6bCdx6. (PDF 102 kb)
Additional file 4: The ontology used in the ontology-based reasoning is available at https://goo.gl/V2PXKl. (OWL 12 kb)

## Abbreviations

CPG: Clinical practice guideline
EHR: Electronic health records
KB: Knowledge base
KBS: Knowledge-based systems
MKBS: Medical knowledge-based systems
OWL: Web ontology language
PR: Plausible reasoning
RDF: Resource description framework
RDFS: RDF schema
RuleML: Rule markup language
SPARQL: Simple protocol and RDF query language
SPIN: SPARQL inferencing notation
SW: Semantic web
SWRL: Semantic web rule language

## Footnotes

1
https://docs.oracle.com/javase/7/docs/api/java/lang/Math.html
