# Supplementary material for: Semantics-based plausible reasoning to extend the knowledge coverage of medical knowledge bases for improved clinical decision support
Source: BioData Min. 2017 Feb 10;10:7. doi: 10.1186/s13040-017-0123-y (PMC5303296; doi:10.1186/s13040-017-0123-y)
Supplement: Additional file 3: — Details of missing values for each test dataset is available at https://goo.gl/6bCdx6. (PDF 102 kb) [file 13040_2017_123_MOESM3_ESM.pdf]

Attribute	Original Data (# missing)	Datasets with 5% Missing					Datasets with 10% Missing					Datasets with 15% Missing					Datasets with 20% Missing				
		Dataset #5-1*	Dataset #5-2	Dataset #5-3	Dataset #5-4	Dataset #5-5	Dataset #10-1	Dataset #10-2	Dataset #10-3	Dataset #10-4	Dataset #10-5	Dataset #15-1	Dataset #15-2	Dataset #15-3	Dataset #15-4	Dataset #15-5	Dataset #20-1	Dataset #20-2	Dataset #20-3	Dataset #20-4	Dataset #20-5
Age	0	11	5	7	7	3	13	13	16	15	12	25	25	17	16	18	25	37	40	32	31
Sex	0	6	8	7	3	5	11	25	21	16	17	23	20	27	16	21	25	35	25	31	32
Steroid	1	15	7	7	11	5	18	15	10	12	17	21	23	24	25	28	32	30	23	29	34
Antivirals	0	6	14	9	10	8	17	14	13	17	15	27	20	30	26	19	30	23	35	37	30
Fatigue	1	8	7	9	3	10	16	16	13	17	11	26	20	26	23	20	32	34	26	36	20
Malaise	1	10	8	6	9	9	13	18	14	16	22	23	16	21	22	35	31	30	28	36	40
Anorexia	1	8	9	7	12	8	9	19	20	15	14	24	23	22	28	25	26	30	35	34	33
Big Liver	10	12	22	20	24	18	26	25	17	23	24	33	27	30	30	33	42	32	46	33	44
Firm Liver	11	22	16	16	20	19	27	18	34	23	27	35	27	25	38	36	39	43	43	41	40
Spl. Palp.	5	9	9	20	13	12	20	17	22	20	19	23	32	23	25	34	35	36	33	33	31
Spiders	5	13	11	14	10	9	23	24	23	20	16	26	26	27	35	27	38	36	34	46	32
Ascites	5	13	11	11	12	10	18	18	22	22	17	29	29	26	36	23	30	31	29	37	32
Varices	5	12	18	13	12	15	19	18	22	21	23	34	28	29	27	31	40	30	36	36	36
Bilirubin	6	12	15	16	12	13	19	15	21	20	18	24	28	32	29	28	38	46	41	41	32
Alk. Phos.	29	34	38	36	35	32	43	36	40	42	47	47	42	47	48	50	54	58	53	55	58
SGOT	4	14	12	16	7	13	22	22	14	21	22	26	28	27	25	20	35	32	26	33	44
Albumin	16	27	23	21	21	23	36	29	25	31	27	41	38	36	45	29	49	34	40	43	43
ProTime	67	72	72	69	70	72	74	76	76	75	80	76	79	83	88	75	80	81	82	84	73
Histology	0	7	8	7	8	10	17	17	16	13	8	17	21	29	18	20	29	34	38	33	29
Class Label	0	0	0	0	0	0	0	0	0	0	0	0	0	0	0	0	0	0	0	0	0
**Total # missing values**	167	311	313	311	299	294	441	435	439	439	436	580	552	581	600	572	710	712	713	750	714

** **Dataset #x-y**: **x** represents the percentage of missing values and **y** is the number of the dataset.*
